# Phytochemical Characterization and Bioactivity Evaluation of Extracts Obtained via Ultrasound-Assisted Extraction of Medicinal Plant *Phedimus aizoon*

**DOI:** 10.3390/plants13141915

**Published:** 2024-07-11

**Authors:** Jeongho Lee, Minji Kim, Hyerim Son, Seunghee Kim, Sangjin Jo, Agiimaa Janchiv, Soo-Yong Kim, Taek Lee, Hah Young Yoo

**Affiliations:** 1 Department of Biotechnology, Sangmyung University, 20, Hongjimun 2-Gil, Jongno-gu, Seoul 03016, Republic of Korea; 202333021@sangmyung.kr (J.L.); mjsj0507@naver.com (M.K.); 202433022@sangmyung.kr (H.S.); 202334015@sangmyung.kr (S.K.); 2 International Biological Material Research Center, Korea Research Institute of Bioscience and Biotechnology, Daejeon 34141, Republic of Korea; sjjo0925@kribb.re.kr (S.J.); soodole@kribb.re.kr (S.-Y.K.); 3 Ulaanbaatar Science and Technology Park, National University of Mongolia, Ulaanbaatar 13343, Mongolia; agiimaa1114@gmail.com; 4 Department of Chemical Engineering, Kwangwoon University, 20, Kwangwoon-Ro, Nowon-gu, Seoul 01897, Republic of Korea

**Keywords:** medicinal plant, *Phedimus aizoon*, bioactive compound, chromatographic analysis, gallic acid, vanillic acid

## Abstract

*Phedimus aizoon* has been utilized as a medicinal plant in Asia. However, the production of phytochemical-rich extracts from *P. aizoon* and the evaluation of their bioactivity are limited. Herein, phytochemical-rich extracts were prepared by ultrasound-assisted extraction of *P. aizoon*, with a high extraction yield of 16.56%. The extracts contained about 126 mg of phenolics and 31 mg of flavonoids per g of the extracts. The chromatographic analysis (GC-MS and HPLC analyses) identified 19 notable phytochemicals of the extracts from *P. aizoon*, including pentacosane, hexadecanoic acid, gallic acid, vanillic acid, and quercetin. The gallic acid content of the extracts was relatively high at 2.75 mg/g. The identified compounds are known to have various bioactivities, such as antioxidant, antibacterial, and antifungal activities. In fact, the prepared extracts exhibited antioxidant activity at 24–28% of that of ascorbic acid. In addition, it showed antibacterial activity against both *Escherichia coli* (Gram-negative bacteria) and *Staphylococcus aureus* (Gram-positive bacteria). This study highlights that *P. aizoon* deserves attention as a natural bioactive substance and emphasizes the need for applications of the extracts from *P. aizoon*.

## 1. Introduction

*Phedimus aizoon* (formerly known as *Sedum aizoon*) is a perennial herbaceous species of the Crassulaceae family and is widely distributed in China, Korea, Japan, and Mongolia [1]. *P. aizoon* has been recognized as a medicinal and edible plant with various pharmacological effects, such as protecting the cardiovascular system, enhancing immunity, stopping bleeding, and tranquilizing the mind [2,3]. The pharmacological effects of *P. aizoon* (or *S. aizoon*) were introduced in ancient medical classics in China [3]. *P. aizoon* has been reported to contain a variety of bioactive constituents, such as flavonoids, phenolic acids, and triterpenes, which have been considered to contribute to its bioactivities [4,5]. Nevertheless, compared to other medicinal plants, there are few reports on the phytochemical characteristics and bioactivities of *P. aizoon*. For the sustainable utilization of *P. aizoon*, efficient extraction, isolation, identification, and activity evaluation of bioactive compounds are needed.

As one of the strategies for recovering bioactive compounds from plant material, the extraction process (techniques and conditions) can be optimized to maximize the extraction yield of the target bioactive compound [6,7]. Prior to this, the extracts must be systematically characterized in terms of the aspects of their phytochemical compositions and bioactivities [8]. In previous studies, non-conventional techniques, such as ultrasound-assisted extraction (UAE) [9] and microwave-assisted extraction (MAE) [10], have been recommended as efficient methods to extract bioactive compounds including flavonoids from *P. aizoon*. However, the information on the phytochemical profiles and bioactivity of *P. aizoon* extracts obtained by using non-conventional techniques is limited. Previous studies detected bioactive compounds of *P. aizoon* extracts obtained by using a conventional extraction technique known as maceration, using various analyses such as chromatography, infrared spectroscopy, mass spectrometry, and nuclear magnetic resonance, and the following compounds were identified: caffeic acid, gallic acid, vanillic acid, luteolin, quercetin, kaempferol, myricetin, and rutin [3]. In addition, Odeh et al. [11] reported the presence of caffeic acid, catechin, gallic acid, hesperidin, and rutin in *S. rubens*, another species of *Sedum*. These phenolics and flavonoids contribute to the bioactivities and medicinal functions of *P. aizoon*; thus, their contents should be studied after the extraction process.

Herein, we aimed to perform the phytochemical characterization and bioactivity evaluation of *P. aizoon* extracts obtained by UAE. Total phenol and flavonoid contents were quantified, and the phytochemicals were analyzed by gas chromatography–mass spectrometry (GC-MS) and high-performance liquid chromatography (HPLC) analyses. In addition, the bioactivities (antioxidant and antibacterial) of *P. aizoon* extracts were evaluated. The present study aims to contribute to advancing our biochemical knowledge of the medicinal plant *P. aizoon* and, in particular, to explore the potential of its extracts, which are rich in bioactive compounds, from the UAE process.

## 2. Materials and Methods

### 2.1. Chemicals

Ascorbic acid (or vitamin C), Folin–Ciocalteu reagent, NaNO_2_, 2,2′-azino-bis(3-ethylbenzothiozoline)-6-sulfonic acid (ABTS), 2,2-diphenyl-1-picrylhydrazyl radical (DPPH), Na_2_CO_3_, gallic acid, vanillic acid, luteolin, quercetin, catechin, rutin, and hesperidin were acquired from Sigma-Aldrich (St. Louis, MO, USA). Kaempferol and myricetin were acquired from Tokyo Chemical Industry (Tokyo, Japan). Methanol, AlCl_3_, and NaOH were acquired from Duksan Chemical (Ansan, Republic of Korea).

### 2.2. Plant Material

**Figure 1:**
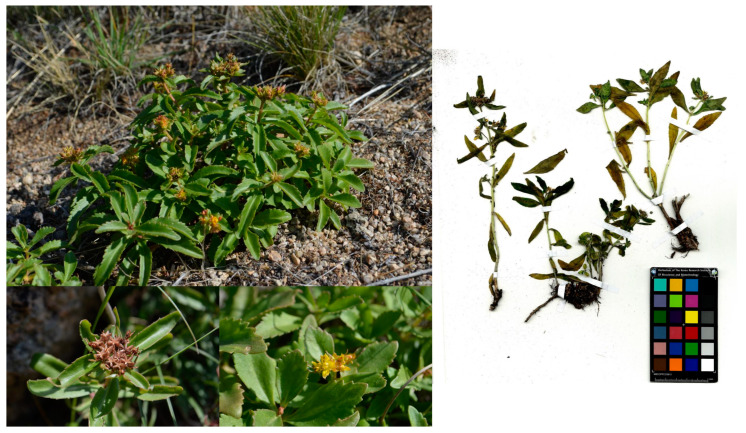
Mongolian plant *Phedimus aizoon* (L.) ‘t Hart.

*Phedimus aizoon* (L.) ‘t Hart was collected in Tsagaannuur district, Huvsugul province, Mongolia, and identified by Dr. Badamtsetseg Bazarragchaa at the National History Museum of Mongolia in August 2015 (Figure 1). A voucher specimen (accession number KRIB 0070462) of the retained material is preserved at the herbarium of KRIBB.

### 2.3. Ultrasound-Assisted Extraction (UAE) of Bioactive Compounds from Phedimus aizoon

A mixture of leaves, shoots, and flowers of *P. aizoon* was used for the extraction. The plant materials were dried in the shade and powdered (water content: 19.1%). In general, methanol is commonly used in the extraction process of bioactive compounds, including flavonoids; thus, methanol was used as an extraction solvent [10,12]. A total of 55 g of plant material was immersed in 1 L of methanol (99.9%) and extracted using an ultrasonic extractor (SDN-900H, SD-ULTRASONIC, Co., Ltd., Seoul, Republic of Korea) for 30 cycles (40 KHz; 1500 W; 15 min sonication and 120 min standing) at room temperature. After extraction, the extracts were filtered and then concentrated at 45 °C using a rotary evaporator (N-1000SWD, EYELA, Tokyo, Japan) and freeze-dried.

### 2.4. Phytochemical Analysis

#### 2.4.1. Estimation of Phenolic Content and Flavonoid Content

The total phenolic and flavonoid contents of the extracts from *P. aizoon* were measured according to our previous study [12]. For the estimation of total phenolic content, 10 μL of the sample (i.e., the extracts dissolved in methanol), 790 μL of water, and 50 μL of Folin–Ciocalteu reagent were mixed and incubated at 30 °C. After 8 min, 150 μL of Na_2_CO_3_ (20% solution) was added and incubated at 25 °C. After 60 min, the absorbance (*A*) of the final mixture was recorded at 700 nm. The data are shown as mg caffeic acid equivalent (CAE) per g extracts. For the estimation of total flavonoid content, 50 μL of the sample and 30 μL of NaNO_2_ (5% solution) were mixed and incubated at 25 °C. After 6 min, 50 μL of AlCl_3_ (10% solution) was added and incubated at 25 °C. After 5 min, 300 μL of NaOH (1 M solution) and 1000 μL of water were added and incubated at 25 °C. After 15 min, the absorbance (*A*) of the final mixture was recorded at 510 nm. The data are shown as mg catechin equivalent (CE) per g extracts.

#### 2.4.2. GC-MS Analysis

GC–MS analysis was performed according to previous studies [13,14] with minor modifications. The extracts from *P. aizoon* were dissolved in methanol, with a final concentration of about 5 μg/mL. The analysis was performed using GC 7890A, 5975C (Agilent, Santa Clara, CA, USA), equipped with a DB 5MS column (0.25 mm × 0.25 µm, 30 m length) with a single quadrupole detection system and an injection volume of 1 µL. The detailed conditions were set as follows: run time, 34 min; oven temperature, initially 60 °C and increased to 280 °C at a rate of 10 °C/min. The National Institute of Standards and Technology (NIST) Library database was used to identify compounds in *P. aizoon* extracts.

#### 2.4.3. HPLC Analysis

For the quantification of several phytochemicals in the sample (i.e., the extracts dissolved in methanol), the HPLC–diode array detector (DAD) (G7117C, Agilent, Santa Clara, CA, USA) analysis was conducted according to our previous study [12]. An INNO column C18 (5 μm, 4.6 mm × 250 mm) was used for analysis and the column temperature was set to 25 °C. A volume of 5 μL of the sample was injected, and the gradient mode was set as follows (solvent A, 0.03% phosphoric acid in deionized water; solvent B, acetonitrile): 0 min, 90% A; 15 min, 80% A; 28 min, 60% A; 36 min, 25% A; 38 min, 90% A; and 50 min, 90% A. The monitoring wavelengths were 250 nm (for vanillic acid), 280 nm (for caffeic acid and gallic acid), and 360 nm (for luteolin, quercetin, and kaempferol). The standard materials of the phytochemicals were selected based on a previous study, which reported the major phytochemicals of *P. aizoon* [3].

### 2.5. Assessment of Antioxidant and Antibacterial Activity

#### 2.5.1. Antioxidant Activity

Various concentrations of the extracts (20–60 and 240–280 µg/mL) or ascorbic acid (7–11 and 50–90 µg/mL) were prepared and then used to evaluate their antioxidant activity. The ABTS method can be used to evaluate the antioxidant activity of hydrophilic and lipophilic compounds, whereas the DPPH method can only evaluate hydrophobic compounds. Therefore, the antioxidant activity of the extracts was assessed based on two methods [12,15]. After the ABTS cation radical scavenging reaction of *P. aizoon* extracts, the absorbance (*A*) was recorded at 734 nm. The radical scavenging activity was calculated using Formula (1). After the DPPH free radical scavenging reaction of *P. aizoon* extracts, the absorbance (*A*) was recorded at 517 nm. The radical scavenging activity was calculated using Formula (1).

Radical scavenging activity (%) = (1 − *A*_sample_/*A*_control_) × 100, (1)

where *A* is the absorbance at 734 nm or 517 nm (blank solution: methanol). The data are expressed as the concentration of *P. aizoon* extracts required to scavenge 50% of the initial radicals (i.e., IC_50_).

#### 2.5.2. Antibacterial Activity

The antibacterial activity of *P. aizoon* extracts was evaluated according to Dutta et al. [16] with minor modifications. *Escherichia coli* and *Staphylococcus aureus* were cultured in a nutrient broth medium for 24 h. The cultured broth with an optical density at 600 nm (OD_600 nm_) = 0.1 was inoculated into 5 mL of the nutrient broth medium. *P. aizoon* extracts were not included in the control group, and the extracts were added to the experimental group (final concentration of extracts in medium = 1 g/L). The broth was incubated at 37 °C and 180 rpm for 1 day, and the OD_600 nm_ of the broth cultured for 4, 8, and 24 h was measured. In addition, to confirm viable cells, the broth cultured for 24 h was spread on a nutrient agar plate and incubated at 37 °C for 1 day.

### 2.6. Statistical Analysis

The extraction process was performed in triplicate to determine the extraction yield from *P. aizoon*. Phenol and flavonoid content determination and bioactivity (antioxidant and antibacterial activity) evaluation were performed in triplicate. All data are expressed as the mean ± standard deviation. Chromatographic analysis was performed once. Significant differences in antioxidant and antibacterial activities between the control (or reference group) and the experimental group were identified by a *t*-test with Sigmaplot (version 12.5; Systat Software Inc., San Jose, CA, USA).

## 3. Results

### 3.1. Analysis of Phytochemicals in Extracts from Phedimus aizoon

Ultrasound-assisted extraction was performed to produce extracts from *P. aizoon*. As a result, the extraction yield was determined to be 16.56 ± 0.39% (dry weight basis) (Table 1). The content of phenolics and flavonoids in the extracts plays an important role in their bioactivity, and thus, the analysis was carried out on *P. aizoon* extracts. The total phenolic content and the total flavonoid content of the extracts from *P. aizoon* were determined to be 126.3 ± 2.2 mg caffeic acid equivalent (CAE)/g extracts and 31.0 ± 0.5 mg catechin equivalent (CE)/g extracts, respectively, which means that about 12.6% and 3.1% of the extracts were phenolics and flavonoids (Table 1). Considering the extraction yield (16.56%) and the phenol and flavonoid content in the extracts, the yield of phytochemicals recovered from 100 g of *P. aizoon* was estimated to be about 2.09 g phenol and 0.51 g flavonoid/100 g biomass (dry weight basis).

#### 3.1.1. GC-MS Analysis

GC-MS analysis was performed to extend the phytochemical characterization of the extracts from *P. aizoon* to volatile compounds. As a result, the presence of at least 13 compounds was identified by searching in NIST Library (Table 2). Decane, undecane, dodecane, benzene, 1,3-bis(1,1-dimethylethyl)-, cyclohexasiloxane, dodecamethyl-, tetradecane, 2,4-di-tert-butylphenol, and hexadecanoic acid, methyl ester had a quality of at least 90%. The other five compounds (Dodecane, 1-iodo-, Cycloheptasiloxane, tetradecamethyl-, Pentacosane, 10-Methylnonadecane, and Hentriacontane) were identified as having a quality (library match) of at least 80%.

#### 3.1.2. HPLC-DAD Analysis

**Figure A1:**
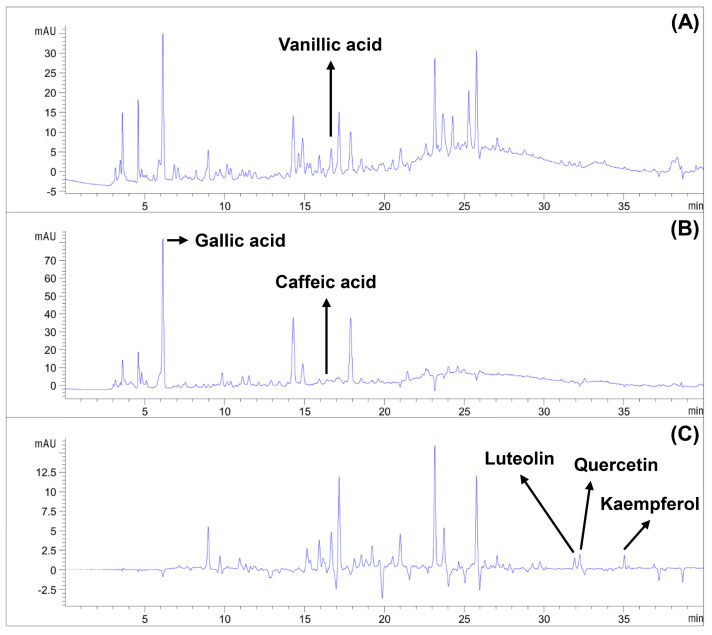
High-performance liquid chromatography chromatograms for *Phedimus aizoon* extracts. The detector monitored at wavelengths of 250 nm (**A**), 280 nm (**B**), and 360 nm (**C**).

Major bioactive compounds of *P. aizoon*, including phenolics and flavonoids, were analyzed by HPLC-DAD analysis (Figure A1). As known compounds of *Phedimus*, caffeic acid, gallic acid, vanillic acid, luteolin, quercetin, kaempferol, catechin, myricetin, rutin, and hesperidin were analyzed. Table 3 lists the detected compounds and their content in the extracts from *P. aizoon*. The contents (mg/g extracts) were high in the order of gallic acid (2.75), vanillic acid (0.50), quercetin (0.19), caffeic acid (0.13), luteolin (0.12), and kaempferol (0.06). Other bioactive compounds such as catechin, myricetin, rutin, and hesperidin were not detected.

### 3.2. Bioactivities of Extracts from Phedimus aizoon

#### 3.2.1. Antioxidant Activity

The antioxidant activity of *P. aizoon* extracts was evaluated based on radical scavenging activity. Two radicals (ABTS cation and DPPH free) were used for the evaluation, and the results are shown in Table 4. In an ABTS cation radical-based evaluation, 260.0 ± 2.6 µg/mL of the extracts was required for scavenging 50% of the radical. As a reference antioxidant, 72.9 ± 1.0 µg/mL of ascorbic acid was required for scavenging 50% of the ABTS cation radical. In a DPPH free radical-based evaluation, 41.0 ± 2.3 µg/mL of the extracts or 9.8 ± 0.2 µg/mL of ascorbic acid was required for scavenging 50% of the DPPH free radical. The results imply that the activity of the extracts to scavenge ABTS cation and DPPH free radicals is 28% and 24% of that of ascorbic acid, respectively.

#### 3.2.2. Antibacterial Activity

**Figure 2:**
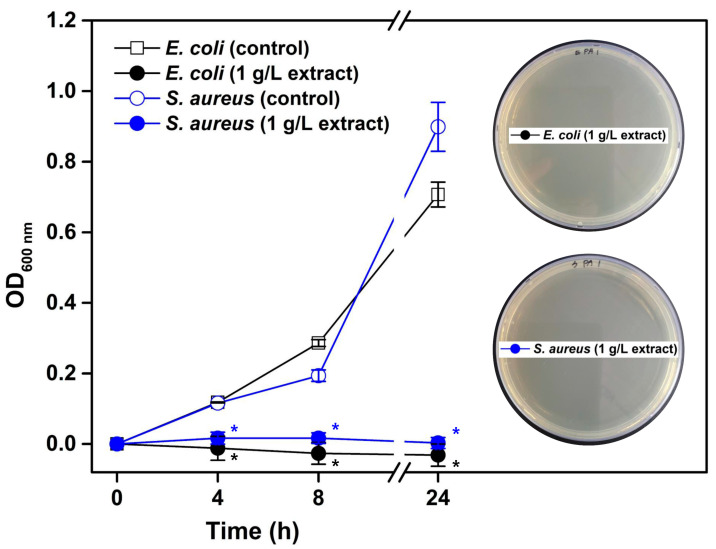
The profiles of the cell growth (OD_600 nm_) of *Escherichia coli* (Gram-negative bacteria) and *Staphylococcus aureus* (Gram-positive bacteria) in nutrient broth media with (experimental group) or without (control group) extracts from *Phedimus aizoon*. Viable cells of both bacteria were not observed in the nutrient agar medium after being spread with the 24 h-cultured broth and incubated, as shown in the right side. The asterisk indicates a significant difference compared to the control group (*p* < 0.05).

The antibacterial activity of the extracts from *P. aizoon* was evaluated based on cell growth profiling. The medium with 1 g/L of the extracts from *P. aizoon* was used for the qualitative evaluation, and the results are shown in Figure 2. In the medium without the extracts from *P. aizoon*, the growth of *E. coli* (Gram-negative bacteria) and *S. aureus* (Gram-positive bacteria) was increased by 24 h. The cell growth (OD_600 nm_) of *E. coli* and *S. aureus* measured at 4, 8, and 24 h was approximately 0.12, 0.29, and 0.71 and 0.12, 0.19, and 0.90, respectively. In contrast, no growth of *E. coli* and *S. aureus* cells was observed in the medium with the extracts from *P. aizoon* at 24 h. Preliminary investigations on the antibacterial activity of the positive control (ampicillin) showed that the minimum inhibitory concentration against *E. coli* and *S. aureus* was about 1.6 and 0.8 mg/L, respectively. Agar plates spread with 24 h-cultured broth of two strains proved that there were no viable cells of the two strains (Figure 2).

## 4. Discussion

The extraction yield in the ultrasound-assisted extraction (UAE) process for *P. aizoon* was about 16.56%, and the total phenol and flavonoid contents of the extracts were 126.3 ± 2.2 and 31.0 ± 0.5 mg/g, respectively (Table 1). This means that the phenol yield and flavonoid yield were 1.69% (i.e., 1692.4 mg/g) and 0.42% (i.e., 415.4 mg/g), respectively. Few previous studies have quantified the total phenol and flavonoid contents (or yields) of *P. aizoon* extracts. Among the conventional extraction techniques, in the maceration process for *P. aizoon*, phenol or flavonoid yield was not available. In the Soxhlet extraction process for *P. aizoon*, the flavonoid yield was determined to be approximately 18.67 mg/g [10]. In the case of non-conventional extraction processes, the flavonoid yield was achieved at approximately 24.87 mg/g under optimal MAE conditions (20 min, 57 °C, 20 mL solvent/g solid, and 80.6% ethanol) [10]. Yin and Batbatan [9] reported the optimal conditions of the UAE process for *P. aizoon*, and the flavonoid yield was 10.77 mg/g under the optimal conditions (1 g solid/55 mL solvent, 60% ethanol, 45 °C, 25 min, and 150 W ultrasound power). The relatively high flavonoid yield in the present study can be attributed to the sufficient time (3 days in total) of the UAE process (see Section 2.3). Further studies should propose optimized UAE conditions to obtain high yields in a shorter time.

The results of the GC-MS analysis identified various phytochemical and bioactive compounds in the extracts from *P. aizoon* (Table 2). Cyclohexasiloxane, dodecamethyl- has been reported to have hair-conditioning, antimicrobial, antiseptic, and skin-conditioning activities [17]. Cycloheptasiloxane, tetradecamethyl- also has various bioactivities such as antibacterial, immunomodulatory, antitumor, antifungal, and antifouling activities [17]. In addition, the biological activities of various phytochemicals detected by GC-MS analysis have been reported earlier: tetradecane, antioxidant and antifungal activities [18]; pentacosane, antibacterial activity [19]; 2,4-Di-tert-butylphenol, antioxidant, anti-inflammatory, antibacterial, antiviral, and antifungal activities [20]; hexadecanoic acid, methyl ester, antioxidant and antibacterial activities [21]. As a result of the HPLC-DAD analysis, various phytochemical and bioactive compounds in the extracts from *P. aizoon* were quantified (Table 3). Overall, compounds that previous studies have identified as present in *P. aizoon* were detected. Lin et al. [22] identified eleven compounds of *P. aizoon* by IR, MS, and NMR, and caffeic acid, gallic acid, vallinic acid, luteolin, quercetin, and kaempferol were contained in the detected compounds. Xu et al. [23] also identified gallic acid, quercetin, and kaempferol in *P. aizoon* extracts using UPLC-DAD-Q-TOF-MS. Phenolic acids (caffeic acid, gallic acid, and vanillic acid) and flavonoids (luteolin, quercetin, and kaempferol) contribute to a variety of biological activities, including antioxidant, antibacterial, anti-inflammatory, and anti-cancer [24]. Thus, *P. aizoon* extracts containing these phenolics and flavonoids were expected to exhibit antioxidant and antibacterial activities.

In fact, *P. aizoon* extracts showed antioxidant and antibacterial activities (Table 4 and Figure 2). Qi et al. [25] optimized process conditions to maximize the flavonoid extraction yield from *P. aizoon* and evaluated the ABTS cation and DPPH free radical scavenging activities of the extracts based on their flavonoid concentration: (1) *P. aizoon* extracts and vitamin C (i.e., ascorbic acid) at 0.3 mg/mL scavenged about 96.2% and 99.0% of ABTS cation radicals, respectively; (2) *P. aizoon* extracts and vitamin C at 2.0 mg/mL scavenged about 85.9% and 98.8% of DPPH free radicals, respectively [25]. In this study, the IC_50_ of the prepared *P. aizoon* extracts was 260 µg/mL and 41 µg/mL for ABTS cation and DPPH free radicals, respectively (Table 4). Nevertheless, due to the limited number of previous studies evaluating the antioxidant activity of *P. aizoon* in vitro and the different criteria for the concentration (extract or flavonoid), the antioxidant properties of *P. aizoon* extracts as natural antioxidants should be further evaluated.

*P. aizoon* extracts showed antibacterial activity against both *E. coli* and *S. aureus* (Figure 2). Several previous studies have reported the antibacterial activity of *P. aizoon* extracts against the Gram-negative bacteria *Aeromonas* [26], *Shewanella putrefaciens* [27], and *Pseudomonas fragi* [5]. In contrast, Oyungerel and Kim [28] reported that *P. aizoon* extracts showed antibacterial activity against *S. aureus*, but not against *E. coli*. Based on these two previous studies and the current study, it is assumed that *P. aizoon* extracts have antibacterial activity against both Gram-negative and Gram-positive bacteria. The antimicrobial activity of *P. aizoon* extracts was summarized as the disruption of cellular metabolic processes, redox homeostasis, and the cause of damage to the bacterial surface and the membrane [3]. The treatment of Gram-negative bacteria *Pseudomonas fragi* with flavonoids from *P. aizoon* resulted in the disruption of the intracellular sulfate assimilation pathway and disturbances in glutathione redox homeostasis [5]. Therefore, the antibacterial activity of the extract prepared in this study against *E. coli* could be attributed to these mechanisms. Until now, regarding the antibacterial activity of *P. aizoon* extracts, advanced research (antibacterial mechanism, application as natural replacement for preservatives) has been focused on Gram-negative bacteria [5,26,27]; thus, further studies should be conducted in depth on Gram-positive bacteria. In addition, for industrial production of *P. aizoon* extracts, an evaluation of the usability of solvents that are generally recognized as safe (GRAS) and optimization of the extraction process can be performed [29]. The produced *P. aizoon* extracts are expected to be utilized as bioactive compounds for the fabrication of antioxidant and antibacterial polymers and nanocomposites [30,31].

## 5. Conclusions

In conclusion, this study established the composition and bioactivity of phytochemical-rich *P. aizoon* extracts with high potential as natural bioactive substances. By applying the ultrasound-assisted extraction (UAE) process, the extracts were produced from *P. aizoon* with a yield of about 16.56%, of which more than 12.6% were phenolic compounds. Well-known bioactive compounds such as cyclohexasiloxane, dodecamethyl-, tetradecane, pentacosane, and hexadecanoic acid were identified, and six compounds were quantified, including gallic acid, vanillic acid, and quercetin. Of these, gallic acid was present in the highest amount at 2.75 mg/g extract. The prepared *P. aizoon* extracts showed outstanding ABTS cation radical scavenging activity (IC_50_: 260.0 ± 2.6 µg/mL) and DPPH free radical scavenging activity (IC_50_: 41.0 ± 2.3 µg/mL), which were 28% and 24% of the activity of ascorbic acid. Furthermore, the antibacterial activity of *P. aizoon* extracts was effective against both Gram-negative and Gram-positive bacteria. This study provides useful information for further phytochemical and biological studies on *P. aizoon* regarding the aspects of in vivo bioactivity, antibacterial mechanism, and applications.

## Figures and Tables

**Table 1:** The extraction yield for *Phedimus aizoon* in the ultrasound-assisted extraction process and the contents of bioactive compounds in the extracts.

Extraction Yield	Total Phenol Content	Total Flavonoid Content
16.56 ± 0.39%	126.3 ± 2.2 mg CAE/g extracts	31.0 ± 0.5 mg CE/g extracts

CAE, caffeic acid equivalent; CE, catechin equivalent.

**Table 2:** Profiles of phytochemicals of extracts from *Phedimus aizoon* in GC-MS analysis.

No.	Compound ^1^	RT (min)	Molecular Weight	Formula	Quality
1	Decane	6.290	142	C_10_H_22_	91
2	Undecane	7.886	156	C_11_H_24_	90
3	Dodecane	9.423	170	C_12_H_26_	94
4	Benzene, 1,3-bis(1,1-dimethylethyl)-	10.192	190	C_14_H_22_	95
5	Dodecane, 1-iodo-	10.522	296	C_12_H_25_I	80
6	Cyclohexasiloxane, dodecamethyl-	10.807	444	C_12_H_36_O_6_Si_6_	91
7	Tetradecane	12.242	198	C_14_H_30_	97
8	Cycloheptasiloxane, tetradecamethyl-	13.026	519	C_14_H_42_O_7_Si_7_	87
9	Pentacosane	13.377	352	C_25_H_52_	86
10	2,4-Di-tert-butylphenol	13.611	206	C_14_H_22_O	93
11	10-Methylnonadecane	13.948	282	C_20_H_42_	80
12	Hentriacontane	15.896	437	C_31_H_64_	86
13	Hexadecanoic acid, methyl ester	18.276	270	C_17_H_34_O_2_	97

^1^ Compounds with a quality of at least 80% are listed.

**Table 3:** Major phenolic acids and flavonoids in extracts from *Phedimus aizoon* in HPLC analysis.

**Compound**	Caffeic acid	Gallic acid	Vanillic acid	Luteolin	Quercetin	Kaempferol
**Content (mg/g Extracts)**	0.13	2.75	0.50	0.12	0.19	0.06

**Table 4:** ABTS cation and DPPH free radical scavenging activity of *Phedimus aizoon* extracts. The asterisk indicates a significant difference compared to the reference antioxidant (*p* < 0.05).

Sample	Radical Scavenging Activity (IC_50_, µg/mL)	
	ABTS Cation Radical	DPPH Free Radical
*Phedimus aizoon* extracts	260.0 ± 2.6 *	41.0 ± 2.3 *
Ascorbic acid (reference)	72.9 ± 1.0	9.8 ± 0.2

## Data Availability

The original contributions presented in this study are included in the article; further inquiries can be directed to the corresponding author.
